# Dabigatran versus vitamin K antagonists for atrial fibrillation in clinical practice: final outcomes from Phase III of the GLORIA-AF registry

**DOI:** 10.1007/s00392-021-01957-1

**Published:** 2022-03-16

**Authors:** Menno V. Huisman, Christine Teutsch, Shihai Lu, Hans-Christoph Diener, Sergio J. Dubner, Jonathan L. Halperin, Chang-Sheng Ma, Kenneth J. Rothman, Ragna Lohmann, Venkatesh Kumar Gurusamy, Dorothee B. Bartels, Gregory Y. H. Lip, for the GLORIA-AF Investigators

**Affiliations:** 1 Department of Thrombosis and Hemostasis, Leiden University Medical Center, Albinusdreef 2, Leiden, the Netherlands; 2 Department of CardioMetabolism and Respiratory Medicine, Boehringer Ingelheim International GmbH, Ingelheim, Germany; 3 Biostatistics, Biogen, Cambridge, MA USA; 4 Institute for Medical Informatics, Biometry and Epidemiology, Medical Faculty of the University of Duisburg-Essen, Essen, Germany; 5 Cardiology Department, Electrophysiology Service, Clínica y Maternidad Suizo Argentina, Buenos Aires, Argentina; 6 The Cardiovascular Institute, Icahn School of Medicine at Mount Sinai, New York, NY USA; 7 Cardiology Department, Atrial Fibrillation Center, Beijing Anzhen Hospital, Capital Medical University, Beijing, China; 8 RTI Health Solutions, Research Triangle Park, NC USA; 9 Clinical Operations, Boehringer Ingelheim Pharma and Co. KG, Ingelheim, Germany; 10 Global Epidemiology, Boehringer Ingelheim International GmbH, Ingelheim, Germany; 11 Institute for Epidemiology, Social Medicine and Health System, Hannover Medical School, Hannover, Germany; 12 Liverpool Centre for Cardiovascular Science, University of Liverpool and Liverpool Heart and Chest Hospital, Liverpool, UK

**Keywords:** Anticoagulation, Atrial fibrillation, Dabigatran, Vitamin K antagonist, Stroke prevention

## Abstract

**Background:**

Prospectively collected, routine clinical practice-based data on antithrombotic therapy in non-valvular atrial fibrillation (AF) patients are important for assessing real-world comparative outcomes. The objective was to compare the safety and effectiveness of dabigatran versus vitamin K antagonists (VKAs) in patients with newly diagnosed AF.

**Methods and results:**

GLORIA-AF is a large, prospective, global registry program. Consecutive patients with newly diagnosed AF and CHA_2_DS_2_-VASc scores ≥ 1 were included and followed for 3 years. To control for differences in patient characteristics, the comparative analysis for dabigatran versus VKA was performed on a propensity score (PS)-matched patient set. Missing data were multiply imputed. Proportional-hazards regression was used to estimate hazard ratios (HRs) for outcomes of interest. Between 2014 and 2016, 21,300 eligible patients were included worldwide: 3839 patients were prescribed dabigatran and 4836 VKA with a median age of 71.0 and 72.0 years, respectively; > 85% in each group had a CHA_2_DS_2_-VASc-score ≥ 2. The PS-matched comparative analysis for dabigatran and VKA included on average 3326 pairs of matched initiators. For dabigatran versus VKAs, adjusted HRs (95% confidence intervals) were: stroke 0.89 (0.59–1.34), major bleeding 0.61 (0.42–0.88), all-cause death 0.78 (0.63–0.97), and myocardial infarction 0.89 (0.53–1.48). Further analyses stratified by PS and region provided similar results.

**Conclusions:**

Dabigatran was associated with a 39% reduced risk of major bleeding and 22% reduced risk for all-cause death compared with VKA. Stroke and myocardial infarction risks were similar, confirming a more favorable benefit-risk profile for dabigatran compared with VKA in clinical practice.

**Clinical trial registration** https://www.clinicaltrials.gov. NCT01468701, NCT01671007.

**Graphical abstract:**

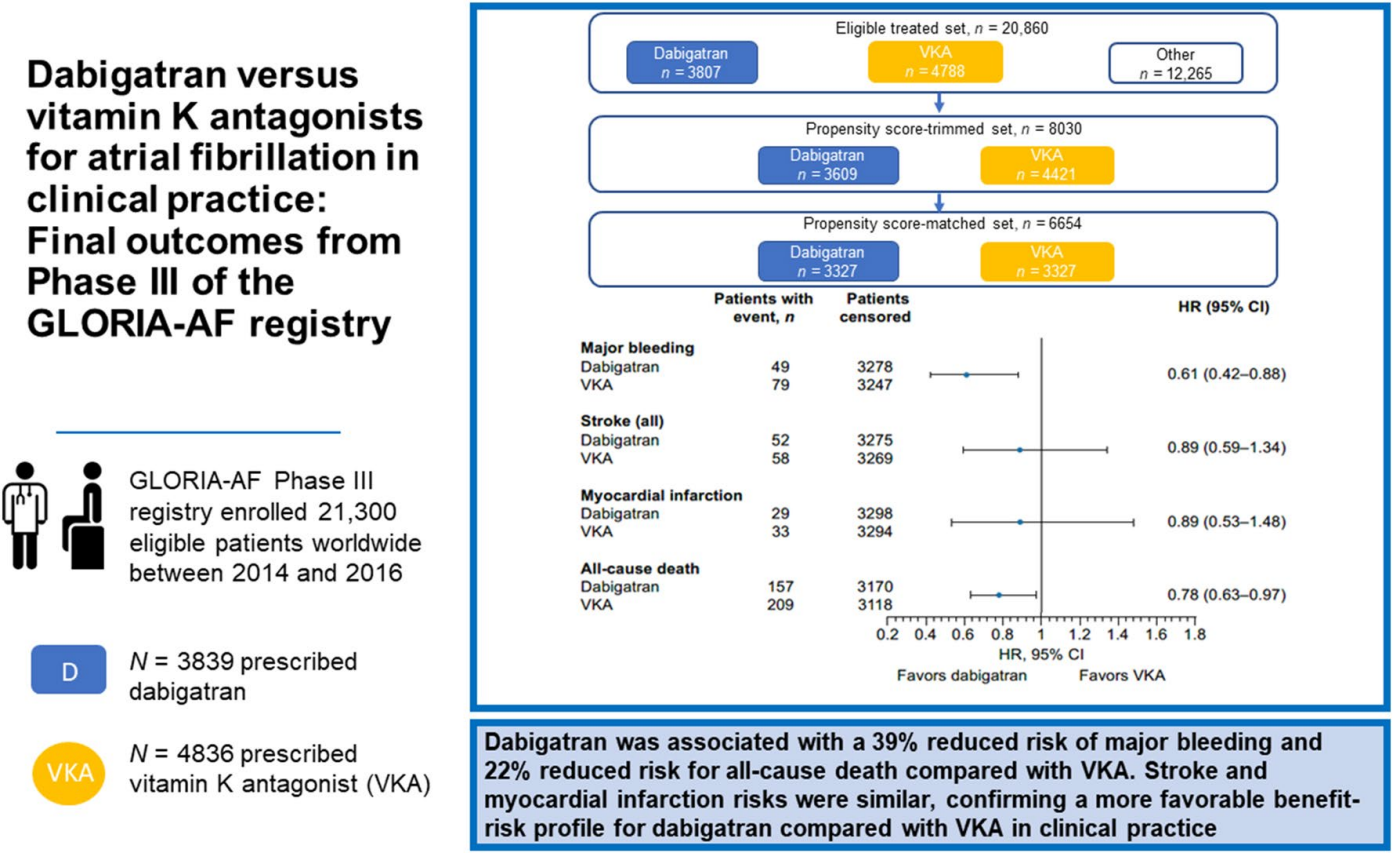

**Supplementary Information:**

The online version contains supplementary material available at 10.1007/s00392-021-01957-1.

## Introduction

Thromboembolic complications are a major cause of morbidity and mortality in patients with non-valvular atrial fibrillation (AF), and while anticoagulation reduces the risk of ischemic stroke, the risk of bleeding is an impediment to broad and sustained implementation [1]. The introduction of the non-vitamin K antagonist oral anticoagulants (NOACs) has profoundly changed anticoagulation management for patients with AF, because NOACs offer greater convenience and net clinical benefit compared with vitamin K antagonists (VKAs) [2]. Four pivotal clinical trials have shown NOACs to be at least non-inferior in the prevention of stroke or systemic embolism in patients with AF and to reduce the risk of intracranial hemorrhage compared with VKA [3–6].

High-quality, practice-based evidence can provide important supplementary data by including patients with characteristics that are under-represented in clinical trials [7]. Retrospective analyses support the safety and effectiveness of dabigatran compared with VKAs or other NOACs in practice [8–10] and a large cohort of Medicare patients exhibited a lower risk of ischemic stroke and intracranial hemorrhage when treated with dabigatran compared with warfarin [10, 11]. To date, prospectively collected observational data are less common.

GLORIA-AF (Global Registry on Long-Term Oral Antithrombotic Treatment in Patients With Atrial Fibrillation) [12] is one of the first large, global, prospective registry programs to provide comparative outcome data for dabigatran versus VKA in routine practice [12, 13]. We describe herein the antithrombotic treatment for stroke prevention in Phase III of GLORIA-AF and report the comparative outcomes of dabigatran versus VKA from the final 3-year follow-up, which was the main objective of GLORIA-AF Phase III.

## Methods

### Study design and setting

**Fig. 1:**
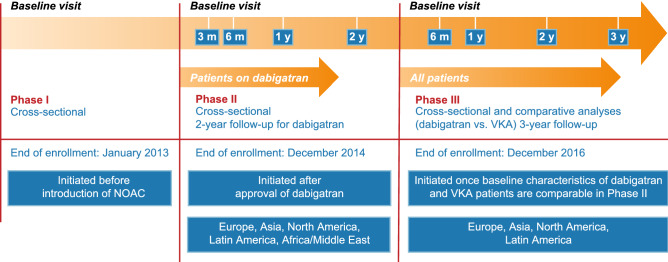
Design of GLORIA-AF. *m*, months*; NOAC* non-vitamin K antagonist oral anticoagulant; *VKA* vitamin K antagonist; *y*, years

GLORIA-AF was an international, multi-center, non-interventional registry program, based on prospectively collected data for patients with newly diagnosed AF. Participating centers were selected to achieve a country-specific balance of health care settings. The three-phased design of the GLORIA-AF Registry Program has previously been published (Fig. 1) [12]. To reduce confounding, Phase III of the program only started once relevant baseline characteristics of patients initiating dabigatran and VKA in Phase II were sufficiently similar to allow for comprehensive comparative analysis, as determined by propensity score (PS) methodology. All patients in GLORIA-AF were managed according to local clinical practice and treatment decisions were solely at the discretion of the treating physician. Patients included in Phase III were followed for 3 years, regardless of prescribed antithrombotic therapy.

GLORIA-AF was conducted in accordance with the principles of Good Clinical Practice and the Declaration of Helsinki and Good Epidemiological Practice and Good Pharmacoepidemiology Practices, and the protocol was approved by the European Medicines Agency and institutional review boards at each participating site. Patients provided written informed consent. An independent, academic steering committee oversaw the design, execution, and study conduct, and was responsible for manuscript development. Extensive measures were undertaken to ensure accurate and complete reporting of outcomes and minimize loss to follow-up. Clinical data and site characteristics were captured using a web-based system over a secure network to ensure confidentiality and data integrity. The Data Sharing Statement is included in the Supplementary Information**.**

### Patients

Physicians were encouraged to enroll consecutive patients who met the inclusion criteria. Eligible patients had a recent diagnosis of AF (< 3 months; except in Latin America, where < 4.5 months was used due to referral patterns), were aged ≥ 18 years, had a risk of stroke (CHA_2_DS_2_-VASc score ≥ 1), and provided written informed consent. Detailed inclusion and exclusion criteria are in Methods 1 in the Supplementary Information.

### Clinical outcomes

Key outcomes (without ranking) were stroke (hemorrhagic, ischemic, and uncertain classification); major bleeding (defined using International Society on Thrombosis and Haemostasis criteria); myocardial infarction; all-cause death; life-threatening bleeding; and the composite of stroke, systemic embolism, myocardial infarction, vascular death, and life-threatening bleeding (definitions in Methods 2 in the Supplementary Information).

### Statistical methods

The Statistical and Epidemiological Analysis Plan was finalized before database lock. Demographics and baseline characteristics were summarized descriptively. Baseline characteristics were compared between dabigatran and VKA patients within different patient sets in terms of standardized differences. For outcome analyses regarding dabigatran and VKAs, missing data for baseline covariates and cause of death were handled using multiple imputation (Methods 3 in the Supplementary Information). All outcome analyses were performed separately for each imputed patient set, and results combined to provide estimates under the missing-at-random assumption. Analyses were performed using SAS® software version 9.4 or later (SAS Institute, Inc., Cary, NC).

**Patient sets** Per protocol, two patient sets were predefined to adjust for unbalanced baseline characteristics in the dabigatran and VKA patient arms.

The PS-trimmed set consisted of a subset of patients obtained after excluding those in the non-overlapping tails of the PS distribution (PS trimming) within each geographic region (Methods 4 in the Supplementary Information). Excluding these patients from the tails of the PS distribution addresses channeling bias and improves the validity of comparisons. The PS-matched set was generated from the PS-trimmed patient set by 1:1 greedy nearest-neighbor matching of dabigatran patients to VKA patients, with a predefined caliper, within region (Methods 4 in the Supplementary Information). Descriptive analyses for the PS-trimmed and PS-matched sets are based on the PS calculated using the first of the multiple imputation patient sets, i.e., the first trimmed and matched sets.

**Clinical outcome analyses** Incidence rates with 95% confidence intervals (CIs) of the key outcome events were calculated for all treatment groups within the eligible patients (Supplementary Table S1). For dabigatran and VKA, incidence rates with 95% CI of the key outcomes were additionally calculated in the PS-trimmed and PS-matched patient sets. The initial analysis comparing effects of dabigatran with VKA was conducted using a multivariable Cox regression model within the PS-trimmed patient set. The model included treatment, age, sex, and risk factors for stroke and bleeding as core variables in the model. Further variables were included based on covariate selection procedures (Methods 5 in the Supplementary Information). Hazard ratios (HRs) with 95% CIs were presented for outcomes considered. The comparative analyses were also conducted in the PS-matched patient set by Cox regression with a shared frailty factor to adjust the matching [14]. Among the matched patients, the degree of balance between dabigatran and VKA for the individual, prespecified covariates (Supplementary Table S2) was assessed. Any covariate with a standardized difference > 10% was considered unbalanced and was also included as a separate variable in the final proportional-hazards regression model. Kaplan–Meier curves were plotted based on the matched patients for a graphical comparison (Methods 6 in the Supplementary Information). Additionally, we conducted a PS stratification analysis, based upon strata formed by deciles of an extended PS and geographic region (Methods 6 in the Supplementary Information).

Longitudinal outcomes were based on an as-treated approach, censoring patients after permanent discontinuation of initial treatment or study termination (definition of permanent discontinuation in Methods 2 in the Supplementary Information).

## Results

### Study patients and baseline characteristics

Between January 2014 and December 2016, 21,591 patients were enrolled at 935 sites from 38 countries, of whom 21,300 were eligible for analysis. Their baseline characteristics are in Supplementary Table S3. Approximately 48% were from Europe, while 24%, 20%, and 8% were from North America, Asia, and Latin America, respectively (Supplementary Fig. S1). Of the 21,300 eligible patients, a total of 17,140 (80.5%) patients completed the planned 3 years of observation time. If a patient did not complete the planned observation time, vital status information was collected where possible. At the end of the study, vital status was available for all but 997 (4.7%) eligible patients who did not complete the planned observation time and had no information on vital status available (i.e., alive or dead).

**Fig. 2:**
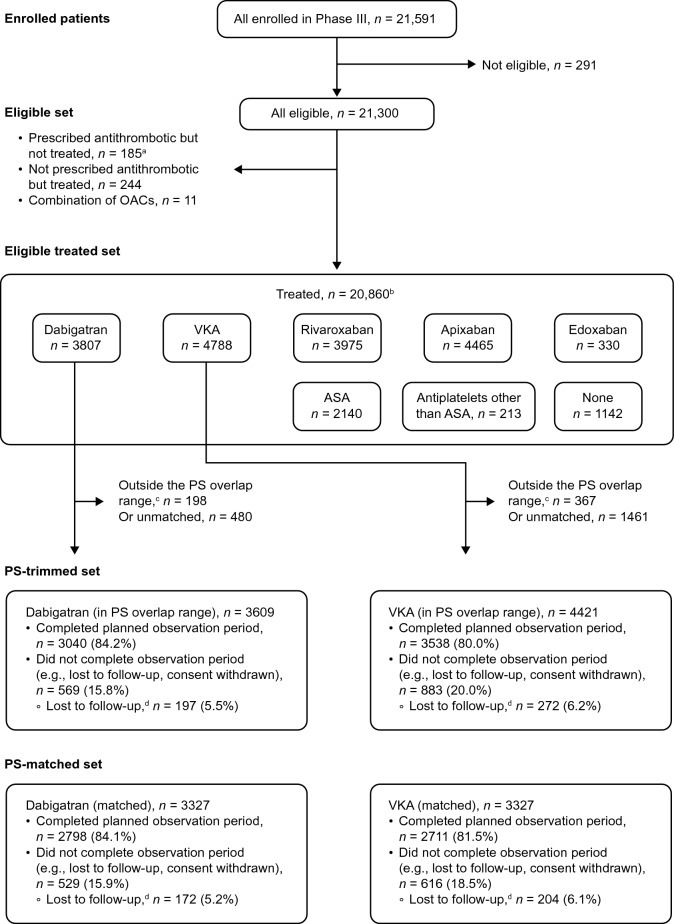
Patient flow. Data are from the patient set determined by the first of the 20 imputed datasets. *ASA* acetylsalicylic acid; *OAC* oral anticoagulant; *PS* propensity score; *VKA* vitamin K antagonist. ^a^Eligible patient set includes patients who were prescribed but did not take the antithrombotic therapies. This includes dabigatran (*n = *32) and VKA (*n = *52). These patients are excluded from the subsequent outcome analyses. ^b^*N*s from individual treatment groups do not add up to the total treated *N* as we do not show all treatments and treatment combinations. ^c^In the dabigatran and VKA groups, patients with a PS less than the 1.5th percentile of the PS distribution for the dabigatran-exposed group and those with PS larger than the 98.5th percentile of the PS distribution for the VKA-exposed group were excluded. ^d^Loss to follow-up is defined as not completed planned observation time and no information on vital status available

The 20,860 “treated” patient population comprised eligible patients who were prescribed an antithrombotic agent and received at least 1 dose of the treatment (19,718 patients) and patients not prescribed an antithrombotic who had no antithrombotic started at baseline (1142 patients). Of the 20,860 patients, 12,577 patients (60.3%) received a NOAC, 4788 (23.0%) VKA, 2140 (10.3%) acetylsalicylic acid alone, 213 (1.02%) antiplatelet other than acetylsalicylic acid, and 1142 (5.5%) had no antithrombotic treatment (Fig. 2).

Table 1 shows baseline characteristics of the eligible patients treated with dabigatran (*n* = 3839) or VKA (*n* = 4836).

**Table 1:** Baseline characteristics of the eligible set treated with dabigatran or VKA

	Eligible patient set^a^		Standardized difference
	Dabigatran *N* = 3839	VKA *N* = 4836	
Age, y			
Median (IQR)	71.0 (64.0–77.0)	72.0 (65.0–79.0)	–0.1064
Mean (SD)	70.1 (10.2)	71.2 (10.3)	
Female sex, *n* (%)	1718 (44.8)	2152 (44.5)	0.0051
Creatinine clearance, mL/min			
Median (IQR)	75.9 (60.2–96.5)	72.2 (53.4–95.2)	0.0769
Mean (SD)	83.5 (117.4)	76.8 (35.4)	
Type of AF, *n* (%)			
Paroxysmal	2082 (54.2)	2174 (45.0)	0.1864
Persistent	1309 (34.1)	1977 (40.9)	− 0.1405
Permanent	448 (11.7)	685 (14.2)	− 0.0744
Medical history, *n* (%)			
Congestive heart failure	695 (18.1)	1284 (26.6)	− 0.2039
History of hypertension	2890 (75.3)	3652 (75.5)	− 0.0055
Diabetes mellitus	828 (21.6)	1233 (25.5)	− 0.0927
Previous stroke	441 (11.5)	462 (9.6)	0.0631
Coronary artery disease	511 (13.3)	916 (18.9)	− 0.1535
Prior bleeding	138 (3.6)	251 (5.2)	− 0.0779
CHA_2_DS_2_-VASc score, mean (SD)	3.1 (1.4)	3.3 (1.5)	–0.1364
HAS-BLED score, mean (SD)	1.2 (0.8)	1.3 (0.9)	0.0207
Previous OAC use within 3 months, *n* (%)	1699 (44.3)	2646 (54.7)	− 0.2103
Chronic concomitant medications, *n* (%)			
Antiplatelet	508 (13.2)	913 (18.9)	− 0.1543
Drugs with higher bleeding risk (HAS-BLED)^b^	569 (14.8)	998 (20.6)	− 0.1527
Region, *n* (%)			
Asia	930 (24.2)	793 (16.4)	0.1955
Europe	2066 (53.8)	2758 (57.0)	− 0.0647
North America	432 (11.3)	736 (15.2)	− 0.1172
Latin America	411 (10.7)	549 (11.4)	− 0.0206
Dabigatran dose, *n* (%)			
150 mg BID	2005 (52.2)	−	−
110 mg BID	1728 (45.0)	−	−
75 mg BID	55 (1.4)	−	−
Other dose	51 (1.3)	−	−

*AF* atrial fibrillation; *BID* twice daily

^a^ Eligible patient set includes patients who were prescribed but not treated with dabigatran (*n* = 32) or VKA (*n* = 52). These patients are excluded from the subsequent outcome analyses

^b^ Concomitant use of drugs associated with higher bleeding risk, as defined in the HAS-BLED score (i.e., antiplatelet agent, Cox-2 inhibitor or other non-steroidal anti-inflammatory drug)

### Baseline characteristics and incidence rates of PS-trimmed patients

In the PS-trimmed patient set, there were 3609 patients treated with dabigatran and 4421 treated with VKAs. Their baseline characteristics are in Supplementary Table S4. In the dabigatran group, 52.6% received 150 mg twice daily (BID), 45.1% received 110 mg BID, and 1.4% 75 mg BID. A total of 3040 (84.2%) dabigatran and 3538 (80.0%) VKA patients completed the 3-year follow-up. The mean (SD) duration of exposure was 25.2 (14.3) months for dabigatran and 24.3 (14.2) months for VKA. The incidence rates for dabigatran and VKA for stroke, major bleeding, and all-cause death are in Table 2.

**Table 2:** Incidence rates of outcomes in the PS-trimmed patient set and the PS-matched patient set treated with dabigatran or VKA^a^

	Propensity-score-trimmed set						Propensity-score-matched set					
	Dabigatran *N* = 3611			VKA *N* = 4413			Dabigatran *N* = 3326			VKA *N* = 3326		
	Pts with event, *n*	PY	IR/100 PY (95% CI)	Pts with event, *n*	PY	IR/100 PY (95% CI)	Pts with event, *n*	PY	IR/100 PY (95% CI)	Pts with event, *n*	PY	IR/100 PY (95% CI)
Major bleeding	51	7440	0.69 (0.51–0.89)	126	8696	1.44 (1.20–1.70)	49	6953	0.70 (0.52–0.91)	79	6526	1.22 (0.93–1.52)
Life-threatening bleeding	35	7444	0.47 (0.32–0.63)	93	8717	1.07 (0.85–1.30)	33	6957	0.48 (0.32–0.65)	59	6537	0.90 (0.66–1.16)
Stroke (all)^b^	57	7425	0.77 (0.58–0.97)	83	8724	0.95 (0.76–1.16)	52	6942	0.74 (0.55–0.95)	58	6541	0.88 (0.64–1.13)
Ischemic stroke	40	7432	0.54 (0.38–0.71)	49	8729	0.56 (0.41–0.73)	36	6949	0.52 (0.35–0.71)	35	6545	0.54 (0.37–0.73)
Hemorrhagic stroke	7	7460	0.09 (0.03–0.16)	28	8750	0.32 (0.21–0.44)	6	6972	0.08 (0.03–0.16)	18	6559	0.28 (0.15–0.43)
Myocardial infarction	30	7449	0.40 (0.27–0.55)	46	8710	0.53 (0.38–0.68)	29	6962	0.41 (0.26–0.57)	33	6528	0.50 (0.32–0.69)
All-cause death^c^	161	7465	2.16 (1.84–2.49)	312	8751	3.56 (3.18–3.94)	157	6976	2.24 (1.89–2.59)	209	6560	3.18 (2.74–3.63)
Composite outcome^d^	164	7396	2.21 (1.88–2.57)	279	8646	3.23 (2.83–3.62)	155	6915	2.24 (1.87–2.60)	184	6484	2.83 (2.41–3.27)

*CI* confidence interval; *IR* incidence rate; *PS* propensity score; *Pts* patients; *PY* patient-years; *VKA* vitamin K antagonist

^a^As the PS was calculated using imputed baseline covariates by multiple imputation, every patient had 20 estimated PSs, leading to 20 different PS-trimmed patient sets. Results presented are based on the average of those sets

^b^Stroke type was classified as uncertain or unknown in 10 patients in the dabigatran group and 6 patients in the VKA group in the PS-trimmed set and in 10 patients in the dabigatran group and 4 patients in the VKA group in the PS-matched set

^c^Unknown cause of death imputed by multiple imputation

^d^Composite outcome: stroke, systemic embolism, myocardial infarction, vascular death, and life-threatening bleeding

### Baseline characteristics and incidence rates of the PS-matched patients

In the PS-matched patient set, 3327 patients were included in each of the 2 treatment groups; their baseline characteristics are shown in Supplementary Table S4.

Specifically, the PS-matched patient set led to the dabigatran and VKA cohorts being closely balanced for all covariates. Table 2 shows incidence rates for dabigatran and VKA in the PS-matched patient set**,** while the incidence rates from the eligible patient population (before multiple imputation) are in Supplementary Table S5.

### Comparative analysis of the PS-trimmed and PS-matched patients

**Fig. 3:**
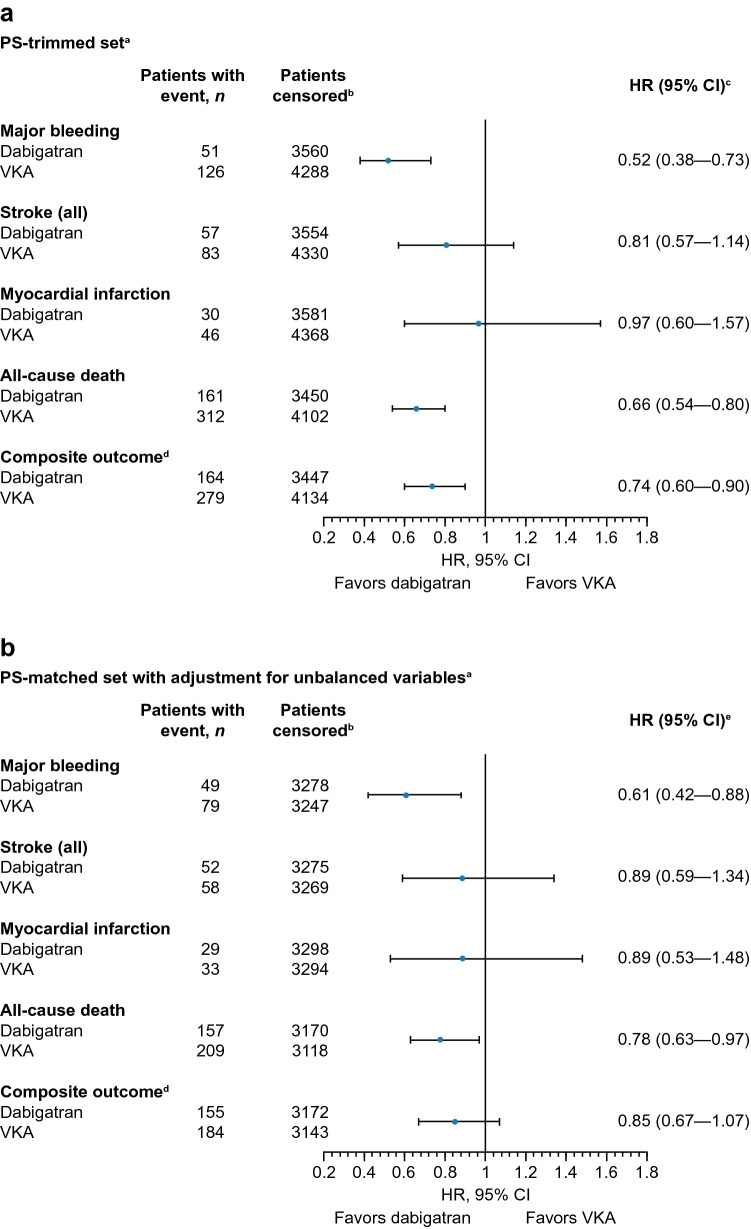
Comparison of outcomes in patients treated with dabigatran or VKA at year 3: (**a**) in the PS-trimmed patient set (primary analysis); (**b**) in the PS-matched patient set with adjustment for unbalanced variables. *CI* confidence interval;* CrCl* creatinine clearance;* HR* hazard ratio;* PS* propensity score; *VKA* vitamin K antagonist. ^a^As the PS was calculated using baseline covariates with missing baseline covariates handled by multiple imputation, every patient had 20 estimated PSs, so there were 20 different PS-trimmed patient sets. Results presented are based on the average of the results from those sets. ^b^Censoring patients after permanent discontinuation of initial treatment or study termination. ^c^Multivariable Cox regression models were used to analyze comparative outcomes of dabigatran versus VKAs, along with a covariate selection procedure (see statistical methods section). ^d^Composite outcome: stroke, systemic embolism, myocardial infarction, vascular death, and life-threatening bleeding, ^e^Treatment, along with unbalanced parameters, are considered in the Cox regression model with a shared frailty factor. CrCl, previous oral anticoagulant use, and type of atrial fibrillation were adjusted in the model, as their standardized difference was > 10% in the matched datasets

Cox regression analysis within the PS-trimmed patient set (Fig. 3A) shows that patients treated with dabigatran had reduced risk for major bleeding (HR 0.52; 95% CI 0.38–0.73), all-cause death (HR 0.66; 95% CI 0.54–0.80), and the composite of stroke, systemic embolism, myocardial infarction, vascular death, and life-threatening bleeding (HR 0.74; 95% CI 0.60–0.90) compared with VKAs. Stroke and myocardial infarction risks were similar (HR 0.81; 95% CI 0.57–1.14 and HR 0.97; 95% CI 0.60–1.57, respectively).

Outcomes for dabigatran versus VKA within the PS-matched set adjusting for unbalanced variables in the Cox model (Fig. 3B) showed that patients treated with dabigatran had reduced risk of major bleeding (HR 0.61; 95% CI 0.42–0.88) and all-cause death (HR 0.78; 95% CI 0.63–0.97) compared with VKA. HR was 0.85 (95% CI 0.67–1.07) for the composite outcome.

**Fig. 4:**
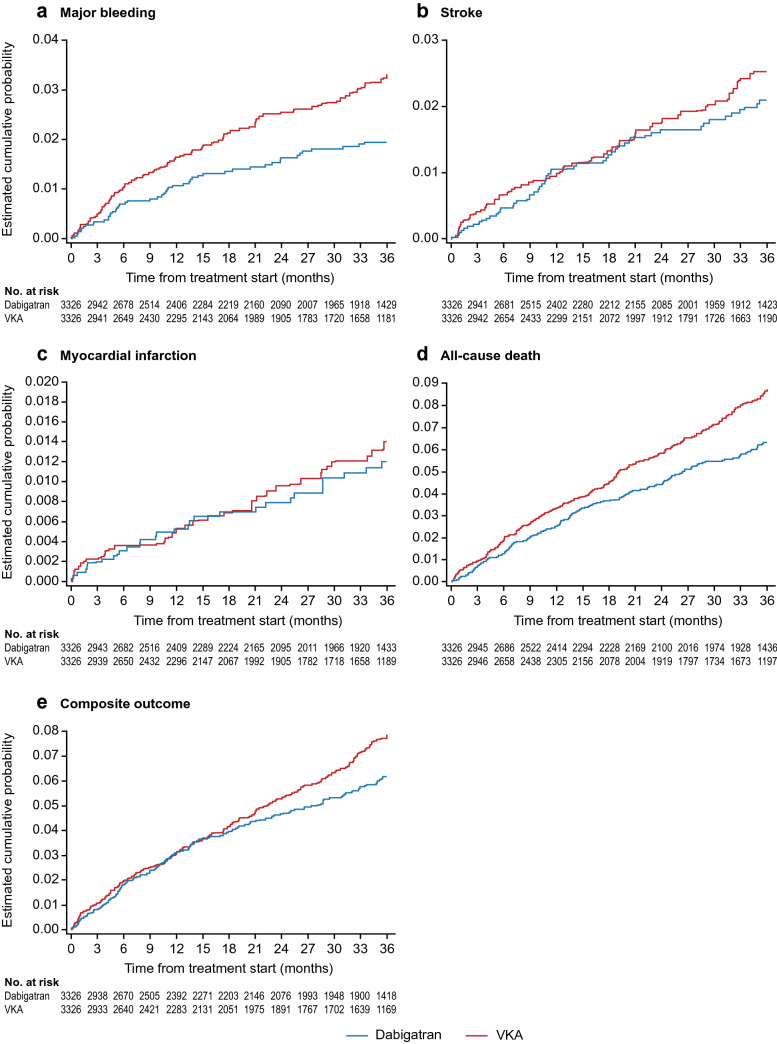
Kaplan–Meier plots of outcomes with dabigatran and VKAs in the PS-matched patient set. *VKA* vitamin K antagonist

Similar risks for stroke and myocardial infarction were observed, which is consistent with the results from the Cox regression analysis within the PS-trimmed set. Figure 4 shows Kaplan–Meier curves of outcomes in the PS-matched set.

Further sensitivity analyses with stratification using a post hoc PS extended sensitivity analysis (Supplementary Fig. S2) showed that the pattern of results was again consistent with the above-described prespecified analyses.

## Discussion

This is the largest prospective, global cohort of consecutive dabigatran- and VKA-treated patients to report long-term follow-up of routine practice-based data comparing the safety and effectiveness of dabigatran with VKAs in patients newly diagnosed with AF. Our principal findings are that, when compared with VKA patients over 3 years of follow-up, dabigatran-treated patients were at reduced risk for major bleeding and all-cause death, with a similar risk of stroke and myocardial infarction. To date, there are only assessments of clinical trial and retrospective analyses comparing dabigatran with VKAs, but GLORIA-AF now provides high-quality data from a prospective global registry.

Prospectively collected, routine, clinical-practice-based data on antithrombotic therapy in non-valvular AF patients are important for assessing real-world comparative outcomes. One specific challenge of comparative analyses in observational studies is differences in patient characteristics based on prescribing information for the drugs of interest as well as physicians’ prescribing preferences that could introduce bias. The design of GLORIA-AF helped to limit bias because Phase III of the study only started in a specific region once relevant baseline characteristics of patients initiating dabigatran and VKA therapy in Phase II showed substantial overlap in the PS distributions [12].

We addressed some of the observed differential prescribing patterns of dabigatran and VKAs using two defined patient sets (PS-trimmed and PS-matched), leading to a better balance for important baseline characteristics. The main drawback of matching is discarding some of the available data; to address this our other analyses, using methods that retain all the patients in the trimmed dataset, preserved precision with otherwise similar findings.

The GLORIA-AF results can be set in context with those using retrospective claims databases to compare dabigatran versus warfarin in various populations. A study of 134,414 elderly US Medicare AF patients treated with dabigatran versus warfarin found lower risk of ischemic stroke (HR 0.80; 95% CI 0.67–0.96), intracranial hemorrhage (HR 0.34; 95% CI 0.26–0.46), and death (HR 0.86; 95% CI 0.77–0.96). Risk of major bleeding was similar (HR 0.97; 95% CI 0.88–1.07) [10]. An analysis of US Department of Defense claims [8] also found risk of stroke (HR 0.73; 95% CI 0.55–0.97), intracranial bleeding (HR 0.49; 95% CI 0.30–0.79), and death (HR 0.64; 95% CI 0.55–0.74) associated with dabigatran treatment were lower compared with warfarin treatment, while major bleeding (HR 0.87; 95% CI 0.74–1.03) was similar. It needs to be considered that these US-based analyses mainly include dabigatran 150 mg BID and a small minority of 75 mg BID, as dabigatran 110 mg BID is not approved in USA. This might also explain the better safety of dabigatran that was observed in GLORIA-AF, in which 45% of dabigatran patients received dabigatran 110 mg BID, a dose that physicians can select for patients at risk of bleeding.

Comparable results, albeit with shorter mean duration of follow-up compared with GLORIA-AF, have also been reported from Danish prescription and patient registries (13 months) [9]. The Danish registry presented data separately for different dabigatran doses. Risk for major bleeding in the VKA-naïve patients was lower versus VKAs for dabigatran 150 mg BID (HR 0.67; 95% CI 0.53–0.85) and similar for dabigatran 110 mg BID (HR 0.91; 95% CI 0.73–1.14); intracranial bleeding risk was lower with both doses of dabigatran versus VKAs (HR 0.32; 95% CI 0.16–0.63 for 150 mg BID and HR 0.31; 95% CI 0.17–0.55 for 110 mg BID) [9].

Two other large registries assessing treatment patterns from routine clinical practice and outcomes in newly diagnosed patients with AF are the Global Anticoagulant Registry in the Field (GARFIELD)-AF and Outcomes Registry for Better Informed Treatment of AF (ORBIT-AF) [15, 16]. In the GARFIELD-AF registry, major bleeding and all-cause mortality were lower with NOACs than VKAs (0.79 [0.70–0.89]; 0.77 [0.61–0.98]), respectively. However, GARFIELD-AF was not a global registry, as North American patients were not included and follow-up was limited to 2 years [16]. Therefore, GLORIA-AF is the only global study providing long-term comparative data complementing the outcomes of pivotal trials through the use of unselected real-world populations and conditions.

GLORIA-AF highlights the value of routine practice data where the clinical outcomes are based on the actual use of dabigatran, including dose selection. The rates of clinical outcomes in this population from the GLORIA-AF study were low and showed a favorable benefit-risk profile for dabigatran compared with VKAs. When comparing the results of GLORIA-AF with the pivotal Phase III trial RE-LY, where patients were randomly assigned to receive either dabigatran 150 mg BID, dabigatran 110 mg BID, or VKAs, dabigatran patients compared with VKA patients in RE-LY had similar or lower rates of major bleeding, while patients taking dabigatran 150 mg BID had lower ischemic stroke rates [6, 17].

The differences between GLORIA-AF and RE-LY are likely due to different study designs such as the randomization of dabigatran doses and differences in the patient populations. For example, GLORIA-AF included only newly diagnosed AF patients, whereas in RE-LY, two-thirds of the patients had pre-existing AF. Furthermore, in GLORIA-AF, the mean CHADS_2_ and HAS-BLED scores were 1.8 and 1.2, compared with mean scores of 2.1 and 1.3, respectively, in RE-LY. Regarding concomitant diseases, while median age, history of hypertension, and diabetes mellitus were similar, history of heart failure was 32% in RE-LY and 21% in GLORIA-AF. Prior stroke/transient ischemic attack history was 20% in RE-LY, while prior stroke/transient ischemic attack/systemic embolism was 14% in GLORIA-AF.

In the RE-LY trial, numerically more myocardial infarction events were observed with dabigatran compared with VKAs [18]. A meta-analysis including > 300,000 patients from retrospective analyses, indicated that the risk for myocardial infarction was similar between dabigatran- and VKA-treated patients [19]. These data are now complemented by the prospective data from GLORIA-AF, in which a similar myocardial infarction risk for dabigatran compared with VKA was also observed. Importantly, follow-up for the GLORIA-AF cohorts was 3 years, the longest described so far in a comparative assessment.

### Limitations and strengths

The generalizability of our study results may be limited by the fact that the study population was restricted to those with a CHA_2_DS_2_-VASc score ≥ 1, though this is similar to other contemporary AF registries. Furthermore, nearly 50% of the cohort was enrolled in Europe. Sites included in GLORIA-AF had to have access to and be able to prescribe both dabigatran and VKAs. Consequently, treatment patterns could be influenced by site selection. To minimize selection bias at the patient level, physicians were encouraged to enroll consecutive consenting patients who met the inclusion criteria.

With no randomization, the study may be subject to confounding by factors not adjusted for in the analysis. Cause of death was unknown in approximately 20% of deaths; for these patients, multiple imputation was used for comparative analysis. Thus, bias may be introduced if the assumptions behind the imputation procedure were not held. To assess the potential impact of this procedure, a sensitivity analysis was performed where unknown death was imputed as vascular cause and a second where unknown death was imputed by non-vascular cause (data on file). The data obtained by these sensitivity analyses were in line with the results observed in the primary analysis.

Strengths of the GLORIA-AF study include the fact that it is the largest prospective global cohort of consecutive dabigatran- and VKA-treated patients reported thus far. Over the 3-year observation period, regular follow-up with physicians, alongside 10% on-site monitoring, and multiple standards for data quality assurance and review ensured event capture. As such, the data quality is strong for an observational setting, with low percentage of eligible patients for whom no information on vital status was available (4.7%). The prospective study design complements other published studies based on retrospective data sources.

## Conclusions

In conclusion, this is the largest, global, prospective cohort of consecutive dabigatran and VKA patients, to report clinical practice-based data comparing the safety and effectiveness of dabigatran with VKAs for stroke prevention in a broad AF patient population. After 3 years' follow-up, the risks of major bleeding and all-cause death for dabigatran were lower compared with VKAs, while the risks for stroke and myocardial infarction were similar. These results confirm a more favorable benefit-risk profile for dabigatran compared with VKAs in routine clinical practice and add to the evidence available for dabigatran in newly diagnosed AF patients, by complementing results from the pivotal RE-LY trial and retrospective analyses performed on various patient populations.

## Supplementary Information

Below is the link to the electronic supplementary material.

Supplementary file1 (DOCX 491 KB)

## Data Availability

Please see Data Sharing Statement in the Supplementary Information.
